# Intrauterine Contraceptive Device Complicated by a Pelvic Abscess: A Case Report

**DOI:** 10.7759/cureus.30728

**Published:** 2022-10-26

**Authors:** Murtaza Syed Hussaini, Niloufar A Tabib, Madison Baker, Malia Omale, Seyed Mohammad Nahidi

**Affiliations:** 1 Internal Medicine, Wyckoff Heights Medical Center, Brooklyn, USA; 2 Internal Medicine, Andrew Taylor Still University (ATSU), Kirksville, USA; 3 Internal Medicine, New York Institute of Technology College of Osteopathic Medicine, Old Westbury, USA; 4 Internal Medicine, St. George’s University, School of Medicine, St. George’s, GRD; 5 Medicine, St. George’s University, School of Medicine, St. George’s, GRD

**Keywords:** postpartum, perforation, long-acting reversible contraception, pelvic inflammatory disease, intrauterine device

## Abstract

Intrauterine devices (IUDs) are a form of long-acting reversible contraception (LARC). As with all medical therapies, their use carries several risks and potential adverse effects. For patients who elect to continue IUD use, pain and irregular bleeding are the most commonly reported complications, but more serious and less common complications include expulsion, contraception failure, pelvic inflammatory disease (PID), and perforation. We report a case where a patient with a history of IUD placement in the immediate postpartum period developed significant complications including multiple intra-abdominal abscesses and pelvic inflammatory disease.

## Introduction

Intrauterine devices (IUDs) are the most common form of reversible contraception. Two types of IUDs are available in the United States: levonorgestrel IUDs and non-hormonal copper IUDs. Complications of IUD can include pain, bleeding, infection, pregnancy, expulsion, and cervical dysplasia. Insertion in the immediate postpartum period is associated with higher rates of complications [1]. A meta-analysis found that perforation risk is independently associated with breastfeeding at the time of insertion and last delivery less than 36 weeks prior to insertion [2]. We report a case of a patient with an IUD placement within a week postpartum who was admitted for an intra-abdominal abscess and pelvic inflammatory disease (PID) resulting from IUD migration and subsequent uterine perforation.

## Case presentation

**Figure 1:**
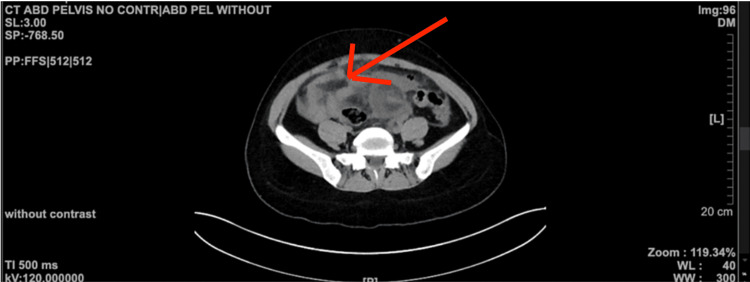
CT scan of the abdomen and pelvis conveying the abscess (red arrow) in the lower abdomen prior to pigtail insertion CT: computed tomography

**Figure 2:**
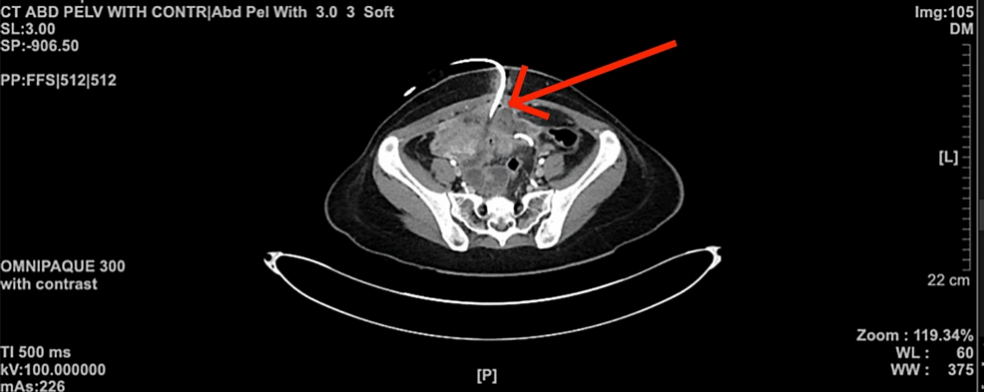
CT scan of the abdomen and pelvis with contrast conveying a pigtail (red arrow) inserted in the region where the abscess is found CT: computed tomography

A 39-year-old female patient with a past medical history of cesarean section (C-section), cholelithiasis, and ovarian cyst presented to our institution complaining of abdominal pain. Upon encounter, her physical examination was significant for generalized abdominal tenderness. The patient’s vitals were significant for fever (102°F) and tachycardia. The C-section surgical scar was clean, and no discharge or signs of infection were noted. Ultrasound of the abdomen was remarkable for a left ovarian mass measuring 9 × 10 × 14 mm. The patient was admitted for sepsis likely secondary to pelvic infection. The patient was initially started on metronidazole, gentamicin, and intravenous fluids. Computed tomography (CT) of the abdomen with contrast conveyed multiple persistent abscesses with the largest abscess measuring 12.1 × 4.9 × 5.8 cm (Figure 1 and Figure 2).

In spite of basic fluid resuscitation and generalized coverage with antibiotics, the patient was noted to be in septic shock, developing tachypnea, tachycardia, and hypoxia requiring 3 L of oxygen via nasal cannula. The patient was transitioned to the intensive critical unit, and aggressive management with broad-spectrum antibiotics including meropenem and vancomycin was commenced with appropriate fluid resuscitation. The patient was closely monitored for the possible requirement of pressor support. The patient underwent diagnostic laparoscopic evaluation and IUD retrieval. Laparoscopy showed diffuse purulent pelvic fluid, migration of the IUD, and intra-abdominal and pelvic adhesions suggestive of Fitz-Hugh-Curtis syndrome. Adhesions were removed during the procedure, and the cysts were aspirated, following which adequate blood, urine, vaginal samples, and the IUD were sent for culture. The patient was then transitioned to a regular medical-surgical floor with pigtail drains accounting for daily drainage of 10-15 cc of purulent fluid. The aspirated fluid from the cyst was positive for *Streptococcus uberis*, which was susceptible to most antimicrobials, and other cultures were negative for any growth. A few days later, the patient underwent laparoscopic-assisted open abscess drainage with a wound vacuum placement. Throughout her stay, the patient received intravenous broad-spectrum antibiotics. As the patient’s clinical symptoms improved, antibiotics were transitioned to oral levofloxacin (750 mg once a day), linezolid (600 mg every 12 hours), and oral fluconazole (100 mg once a day) as recommended by the infectious disease team. The patient was discharged after 16 days of in-patient care.

**Figure 3:**
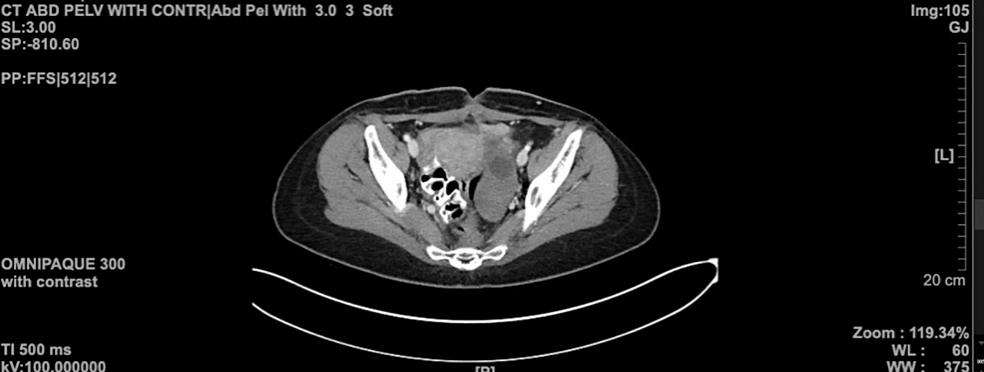
CT scan of the abdomen and pelvis conveying the resolution of the abscess CT: computed tomography

Three weeks after discharge, on a routine follow-up visit, abdominal CT showed complete resolution of the abscesses (Figure 3).

## Discussion

This case illustrates the necessity for prompt and accurate diagnosis of PID to ensure optimal patient outcomes. While intrauterine devices (IUDs) are a generally well-tolerated form of long-acting reversible contraception (LARC), they are not without risks. Pain and irregular bleeding are the most commonly reported reasons among patients who elect to discontinue IUD use. Other rare complications include expulsion, contraception failure, pelvic inflammatory disease (PID), and perforation. Some studies have suggested that immediate postpartum insertion has a higher rate of IUD expulsion [1]. However, the data varies greatly from study to study [3]. Another factor that places patients at an increased risk of IUD expulsion is young age [4]. Young age has also been identified as a significant risk factor for contraception failure in IUD users [5].

Of PID cases, 85% are caused by sexually transmitted cervical pathogens or bacterial vaginosis-associated microbes, and the remaining 15% are caused by respiratory or enteric organisms [6]. The risk of PID in females with IUDs is low [7]. PID is a rare complication of IUD use, even in patients who test positive for gonorrhea and/or chlamydia [8]. PID may, although rarely, lead to the formation of a tubo-ovarian abscess (TOA) [9]. These can be managed with broad-spectrum antibiotics, laparoscopic procedures, and percutaneous drainage [10]. The most common microorganisms in TOAs are thought to be *Escherichia coli*, *Bacteroides fragilis*, *Bacteroides* species, *Peptostreptococcus *species, *Peptococcus *species, and aerobic streptococci [11].

A prospective cohort study with 61,448 patients who had IUD insertions found 81 uterine perforations, none of which resulted in serious illness or injury to intra-abdominal or pelvic structures [2]. The type of IUD (hormonal versus copper) is not thought to significantly alter the risk of perforation [2]. Perforation typically happens at the time of insertion rather than as a result of a delayed migration from the original site [2]. A study that included 18 perforations found that perforation was more likely to occur in patients 0-3 months postpartum than in other patients; the study also found that after six months postpartum, there was no increase in the risk of perforation [12]. However, systematic reviews have not found higher rates of uterine perforation immediately postpartum [13]. We present a case where a young female, who opted for IUD placement within one week postpartum, presented to our emergency department complaining of generalized abdominal pain and was found to have an intra-abdominal abscess. Delay in seeking medical attention upon experiencing such symptoms post-IUD placement can lead to life-threatening consequences. Thus, it is crucial for patients who received an IUD placement to have regular follow-ups within 0-3 months and be educated on when to present to the clinic/emergency department if they feel worsening symptoms.

## Conclusions

Although IUDs are generally well-tolerated, the rare complication of uterine perforation secondary to IUD insertion can result in PID and abscess formation. Without immediate management, this can result in serious sequelae including an acute abdomen, permanent infertility, and death. In this case, our patient was diagnosed with PID, uterine perforation, and an intra-abdominal abscess due to IUD migration and perforation. In such cases, delay in seeking medical attention or early diagnosis may lead to irreversible outcomes. It is crucial for physicians to be aware of this possible differential diagnosis upon encountering patients with a similar history.
